# Epigenetic and Gene Expression Responses of *Daphnia magna* to Polyethylene and Polystyrene Microplastics

**DOI:** 10.3390/molecules30071608

**Published:** 2025-04-03

**Authors:** Hyungjoon Im, Jieun Lee, Jeong-Eun Oh, Jinyoung Song, Sanghyun Jeong

**Affiliations:** 1 Institute for Environment and Energy, Pusan National University, Busan 46241, Republic of Korea; him86@pusan.ac.kr (H.I.); 99atkins07@pusan.ac.kr (J.L.); jeoh@pusan.ac.kr (J.-E.O.); 2 Department of Civil and Environmental Engineering, Pusan National University, Busan 46241, Republic of Korea; 3 Center for Ecotoxicology and Environmental Future Research, Korea Institute of Toxicology, Jinju 52834, Republic of Korea; jinyoung.song@kitox.ac.kr

**Keywords:** nanoplastic, toxicity pathway, DNA methylation, DNA hydroxymethylation

## Abstract

Microplastics (MPs), ubiquitous environmental pollutants, pose substantial threats to aquatic ecosystems and organisms, including the model species *Daphnia magna*. This study examined the effects of polyethylene (PE) and polystyrene (PS) MPs on *D. magna*, focusing on their ingestion, epigenetic alterations, and transcriptional responses. Exposure experiments revealed a concentration-dependent accumulation of MPs, with PS particles showing higher ingestion rates due to their higher density and propensity for aggregation. Epigenetic analyses demonstrated that exposure to PE MPs significantly reduced the global DNA methylation (5-mC) of *Daphnia magna*, suggesting hypomethylation as a potential stress response. Conversely, the DNA hydroxymethylation (5-hmC) of *Daphnia magna* displayed variability under PS exposure. Transcriptional analysis identified a marked downregulation of *Vitellogenin 1* (*v1*) and upregulation of *Ecdysone Receptor B* (*ecr-b*), highlighting the occurrence of stress-related and adaptive molecular responses. These findings enhance our understanding of the molecular and epigenetic effects of MPs on aquatic organisms, offering critical insights for the development of effective environmental management and conservation strategies in the face of escalating MP pollution.

## 1. Introduction

Microplastics (MPs), plastic particles smaller than 5 mm [1], have emerged as significant environmental pollutants, particularly in aquatic ecosystems. MPs originate from diverse sources, including urban runoff, wastewater discharge, and agricultural practices [2,3,4,5,6]. MPs in freshwater environments pose significant threats to various organisms, including *D. magna* [7], which is widely used in ecotoxicological research because of its ecological importance and high sensitivity to environmental changes [8]. *D. magna* is particularly vulnerable to MPs because it filter-feeds on microalgae and other small particles [9,10]. The ingestion of MPs can adversely affect the feeding efficiency, digestion, growth, reproduction, and survival of *D. magna* populations [11,12,13,14], potentially impacting the population dynamics and community structures in aquatic ecosystems [9,15]. These observations highlight the urgent need to investigate the underlying biological mechanisms driving these effects [16,17,18].

While the physiological and individual-level effects of MPs are increasingly well-documented [14], knowledge about their molecular impacts, particularly on gene expression and epigenetic modifications, remains limited. Emerging evidence suggests that MPs may influence the epigenetic landscape of *D. magna* [18]. Recent studies have investigated the role of DNA methylation and hydroxymethylation in responding to environmental stressors, including exposure to MPs [18,19,20].

Global DNA methylation (5-mC) and DNA hydroxymethylation (5-hmC) are pivotal epigenetic modifications involved in regulating gene expression, mediating cellular stress responses, and facilitating adaptation to environmental changes [21,22]. These epigenetic markers have been linked to various types of environmental exposure, including metals such as lead, antimony, and arsenic [23,24], airborne particulate matter (PM10) [25], and crude oil spills [26]. Epigenetic modifications, such as DNA methylation, can regulate the gene expression of an organism without changing the underlying DNA sequence, potentially leading to adaptive or maladaptive stress responses to environmental pollutants [27,28,29]. Examining the global DNA methylation and hydroxymethylation in *D. magna* exposed to MPs could reveal how these pollutants induce epigenetic changes that affect gene expression and compromise organismal health and fitness [30,31]. Moreover, such epigenetic modifications are increasingly recognized as sensitive biomarkers of environmental stress, aiding in the assessment of the impact of MPs on aquatic organisms [32,33]. In this study, the 5-mC and 5-hmC levels of *D. magna* were analyzed to evaluate its epigenetic responses to MP exposure. The ability of *D. magna* to reproduce asexually makes it an invaluable model for studying epigenetic dynamics [34,35].

Despite emerging interest, our understanding of how specific polymer types of MPs, such as polyethylene (PE) and polystyrene (PS), two of the most widely produced and ubiquitously detected polymers [36], affect the epigenome and gene expression in *D. magna* remains incomplete. Comparative studies focusing on these two polymers can help clarify whether their contrasting physicochemical properties drive distinct epigenetic and gene expression responses in *D. magna*.

This study investigates the epigenetic and gene expression responses of *D. magna* to PE and PS MP exposure, focusing on global DNA methylation and hydroxymethylation, as well as stress- and development-related gene expression. The findings aim to provide novel insights into the molecular mechanisms of the effects of MP pollution, enhancing understanding of MPs’ ecotoxicological impacts.

## 2. Results and Discussion

### 2.1. Microplastic Ingestion by D. magna

**Figure 1:**
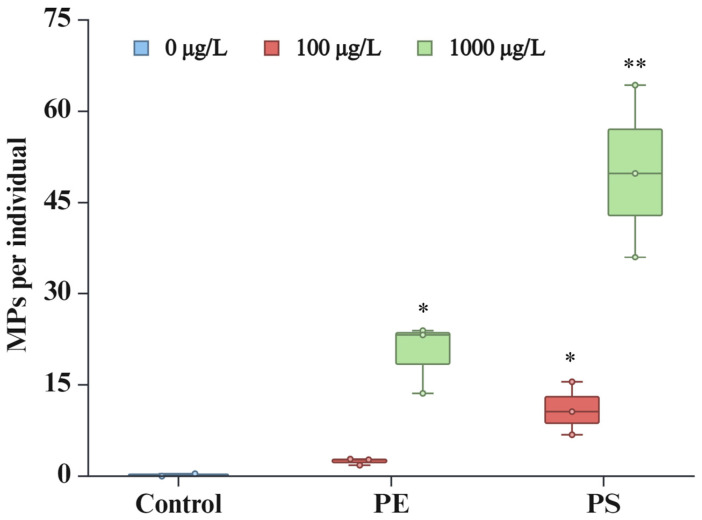
Average number of residual MPs ingested per individual *Daphnia magna* after 7 days of exposure. Measurements were taken at the end of the exposure period (*n* = 3). Each replicate consisted of 8–11 animals. Asterisks indicate statistically significant difference from control group (* *p* ≤ 0.05; ** *p* ≤ 0.001).

Despite regular stirring of the medium during the exposure experiments (every 8 h) to ensure an even distribution of particles, the physiochemical characteristics of the MPs differentially affected their ingestion by *D. magna*. The ingestion of microplastics by *D. magna* was systematically assessed under varying concentrations of PE and PS particles (Figure 1). Under control conditions, minimal ingestion was observed, with an average of 0.18 particles per individual, which is indicative of baseline contamination. Exposure to 100 µg/L of PE resulted in a marked increase in the ingestion rates, with an average of 2.44 particles per individual being observed. This rate escalated significantly at a higher concentration of 1000 µg/L PE, reaching 20.24 particles per individual. A similar trend was observed for PS exposure: at 100 µg/L, the average ingestion rate was 11.03 particles per individual, which increased dramatically at 1000 µg/L, peaking at 50.03 particles per individual.

These findings underline a clear concentration-dependent accumulation of MPs by *D. magna*, with the ingestion rates increasing significantly at higher concentrations of both PE and PS particles. Similar to our results, previous studies have also reported an increase in the accumulation of MPs in *D. magna* with increasing concentration of MPs [37,38,39,40,41].

Another notable observation is that, under identical exposure conditions, the *D. magna* organisms ingested the PS particles at a higher rate than the PE particles. Various physicochemical properties of MPs can influence their ingestion by *D. magna* [42]. In this study, the PS and PE particles had nearly identical shapes and sizes (mean particle sizes of 38–48 µm), so differences in shape likely did not contribute to the observed effects. Instead, the differences in density, buoyancy, and aggregation behavior between PS and PE can be proposed as the primary factors. For instance, PS MPs have a density of approximately 1.05 g/cm^3^ [43], whereas PE MPs have a density of 0.88–0.96 g/cm^3^ [44]. This density difference allows PS particles to remain more evenly suspended in the water column, increasing the likelihood of encounter by *D. magna*, which predominantly feeds below the surface [13,17]. In contrast, PE MPs are more prone to floating on the surface of water, which reduces their availability within the feeding zone of *D. magna*. Similarly, Luangrath et al. (2024) observed higher bioconcentrations of denser MPs (e.g., polylactic acid, ~1.24 g/cm^3^) compared to those of lower-density particles like PE [39].

Another key factor is the propensity of the particle to aggregate. PS particles, due to their higher hydrophobicity, tend to form larger aggregates by interacting strongly with organic matter and microorganisms [45,46,47]. Such aggregation can enhance their effective particle size, making them easier to capture during filter feeding by *D. magna* [48]. Moreover, the interaction of PS MPs with organic matter enhances their bioavailability, as the adsorption of nutrients by the particles and the development of biofilms on them make them more attractive to filter feeders [17,40,49]. In contrast, PE MPs typically remain as smaller, less aggregated particles, which may reduce their likelihood of being consumed by *D. magna* [50,51].

Thus, the higher ingestion rate of PS particles likely stems from their greater suspension stability (due to their higher density) and their enhanced tendency to aggregate, which, together, increase the likelihood of *D. magna* encountering and ingesting these particles. These findings underscore the importance of considering multiple physicochemical properties when evaluating the interaction of MPs with aquatic organisms.

### 2.2. Epigenetic Responses to Microplastic Exposure in D. magna

Global DNA methylation (5-mC) and DNA hydroxymethylation (5-hmC) are pivotal epigenetic modifications involved in regulating gene expression, mediating cellular stress responses, and facilitating adaptation to environmental changes [21,22]. These epigenetic markers have been linked to various types of environmental exposure, including metals such as lead, antimony, and arsenic [23,24], airborne particulate matter (PM10) [25], and crude oil spills [26]. In this study, the 5-mC and 5-hmC levels of *D. magna* were analyzed to evaluate its epigenetic responses to MP exposure. The ability of *D. magna* to reproduce asexually makes it an invaluable model for studying epigenetic dynamics [34,35].

**Figure 2:**
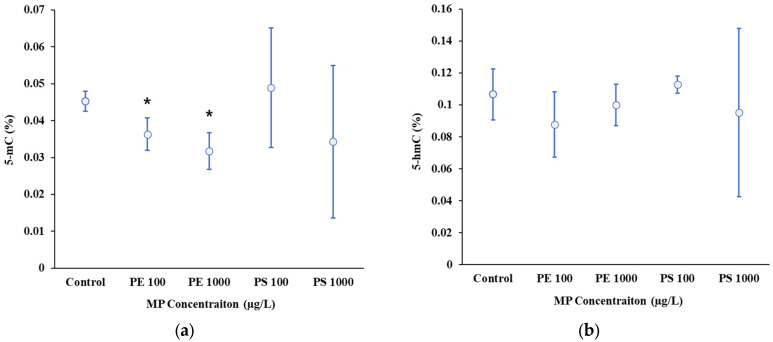
(**a**) Global DNA methylation and (**b**) global DNA hydroxymethylation in *Daphnia magna* exposed to PE and PS MPs at different concentrations (100 µg/L and 1000 µg/L). * Asterisks indicate significant difference from control group (*p* ≤ 0.05).

The results of this study indicate that exposure to MPs, specifically PE and PS, resulted in changes in the DNA methylation levels of *D. magna*. Significant decreases in DNA methylation were observed at higher concentrations of PE (100 and 1000 µg/L), which is indicative of a hypomethylation response (Figure 2a). These finding contrast with the study of Song et al. (2022) [18], which demonstrated no significant changes in global DNA methylation in *D. magna* exposed to PE fragments (16.68 ± 7.04 µm; 4.35 mg/L).

A decrease in DNA methylation, or hypomethylation, is commonly associated with increased gene expression and the activation of stress response pathways (Athanasio et al., 2018) [52]. The hypomethylation observed in the *D. magna* exposed to PE MPs may reflect a stress response mechanism. Similar epigenetic changes in response to environmental pollutants like metals, where the global DNA methylation decreases as organisms reallocate resources to cope with stress, potentially at the expense of growth and reproduction, have been reported [27,53,54].

Understanding the interplay between DNA methylation and oxidative stress is critical to understanding the biological impact of MPs. Oxidative stress, induced by MPs, can influence DNA methylation patterns due to causing a cellular response to environmental stressors. Tang et al. (2019) [55] demonstrated that PE MPs upregulate genes involved in oxidative defense mechanisms, such as thioredoxin reductase (TRxR), which play a critical role in mitigating reactive oxygen species (ROS). This oxidative stress may contribute to the hypomethylation observed in *D. magna* exposed to PE MPs [56].

In contrast to PE, exposure to PS MP particles at concentrations of both 100 and 1000 µg/L did not result in significant changes in the DNA methylation levels observed in this study (Figure 2a). This lack of significant change may result from a counterbalance between hyper- and hypomethylation [28]. Environmental stressors such as MP exposure can trigger transgenerational epigenetic changes in *D. magna*, potentially influencing the resilience of subsequent generations to future challenges. For instance, salinity-induced DNA hypomethylation in *D. magna* has been shown to be inherited by its offspring [28], and other natural stressors can also cause inheritable changes in methylation patterns [30,34]. Similarly, MPs may alter *D. magna*’s DNA methylation, thereby affecting its capacity to adapt and survive in polluted environments [18].

The DNA hydroxymethylation, a distinct epigenetic modification implicated in gene regulation, observed in *D. magna* showed notable variability in the PS 1000 µg/L [57] test (Figure 2b). While not statistically significant, this increased variability suggests potential effects of high PS concentrations on hydroxymethylation patterns. Further investigation is warranted to validate these trends and address experimental variability [58,59].

### 2.3. Transcriptional Response to MP Exposure in D. magna

**Figure 3:**
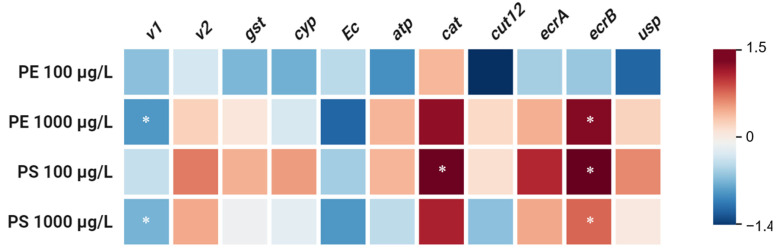
Relative changes in expression of target genes in response to PE and PS MPs exposure at different concentrations (100 µg/L and 1000 µg/L). Vitellogenin 1 (*v1*), vitellogenin 2 (*v2*), glutathione-s-transferase (*gst*), cytochrome P450 (*cyp*), ATPase Na^+^/K^+^ (*atp*), catalase (*cat*) cuticle protein 12 (*cut12*), ecdysone receptor b (*ecr-b*), and universal stress protein (*usp*). Asterisks indicate significant difference from control group (*p* ≤ 0.5).

To elucidate the biological impact of MP exposure, we assessed the transcriptional responses of a panel of genes (*v1*, *v2*, *gst*, *cyp*, *atp*, *cat*, *cut12*, *ecr-b*, *usp*) to the studied MPs. The significant downregulation of *v1* and the upregulation of *ecr-b* were observed in *D. magna* exposed to high concentrations of both PE and PS MPs (1000 µg/L; *p* < 0.05) (Figure 3). These transcriptional changes are indicative of a stress response associated with the presence of high concentrations of MP particles.

The downregulation of the *v1* gene suggests a potential stress response mechanism triggered by MP exposure. The *v2* gene did not show any significant changes. The *v1* gene is noteworthy, as it is associated with physiological processes such as growth and development, and metabolic pathways that regulate the synthesis of vitellogenin (Vtg), which is a precursor to yolk proteins which are critical for reproduction. This downregulation suggests that the *D. magna* may be reallocating resources from reproductive functions to cope with the stress induced by MP exposure. This resource reallocation aligns with the observed decrease in DNA methylation, further supporting the hypothesis of a stress response mechanism that prioritizes survival over growth and reproduction [54]. Moreover, other MP studies have reported concomitant reductions in maternal growth along with the reproductive decline observed herein [16,60,61].

**Figure 4:**
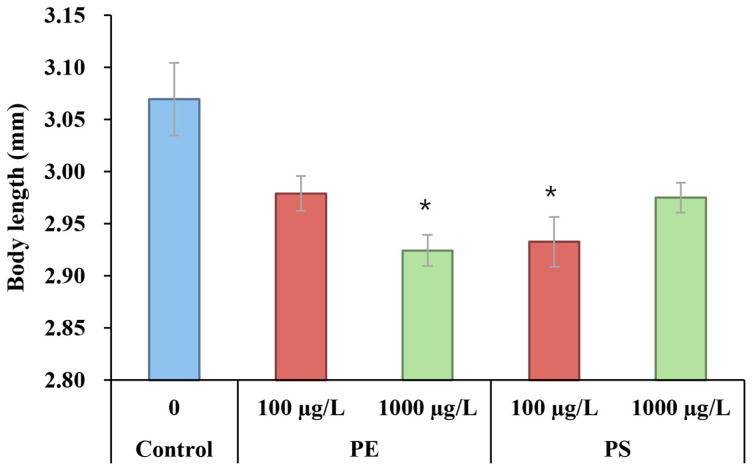
Body length of *Daphnia magna* exposed to PE and PS microplastic particles at different concentrations (100 µg/L and 1000 µg/L) (n = 10). Asterisks indicate statistically significant differences compared to the control group (* *p* ≤ 0.5).

The upregulation of the *ecr-b* gene, which is involved in ecdysone receptor signaling pathways, suggests a potential adaptation or compensatory mechanism that activates in response to MP exposure. Molting, a pivotal process in crustacean growth, is predominantly regulated by the ecdysteroids, with the cytochrome P450 subfamily member Cyp314 playing a critical role in synthesizing the molting hormone ecdysone. This hormone is subsequently converted into its active form, 20-hydroxyecdysone (20E), which is regulated by the ecdysone receptor, *ecr-b*. The *ecr-b* gene plays a crucial role in crustacean development, which includes molting and adaptation to environmental stressors [62]. The upregulation of *ecr-b* observed in the MP-exposed groups suggests an adaptive response aimed at enhancing resilience to MP-induced stress. This response aligns with the critical role of *ecr-b* in facilitating molting and stress adaptation. However, despite this upregulation, the body length in the MP-exposed groups—particularly in the PE 1000 µg/L and PS 100 µg/L groups—was significantly reduced (Figure 4). This discrepancy indicates that, while *ecr-b* upregulation may support molting and stress resilience, the inability of *D. magna* to allocate sufficient resources for growth under MP-induced stress results in a smaller body size. The resource reallocation of *D. magna* may prioritize survival and stress adaptation mechanisms over growth, as observed in previous studies on stress responses in aquatic organisms [63]. In an ecological context, a reduced body size due to microplastics may translate to lower fitness, as evidenced by the fewer offspring and slower population growth observed in the exposed Daphnia. In addition, Genes like *cut12*, which is responsive to 20-hydroxyecdysone (20E), are responsible for producing cuticle proteins that are essential for the formation of new exoskeletons [64], but did not show significant changes in expression in this study. This lack of significant change in the *cut12* expression suggests that, while the molting process is partially supported by *ecr-b* upregulation, other factors or gene expressions that are critical for exoskeleton development may remain unaffected, potentially limiting the organism’s growth and structural integrity.

It is noteworthy that PE and PS may interact with Daphnia at the molecular level in distinct ways: PE exposure caused significant DNA hypomethylation in this study, potentially affecting gene regulation, whereas PS exposure did not show this global methylation loss, instead inducing other cellular stress signals. Such polymer-specific modes of action align with the opposite gene expression trends that were observed, underlining that “all microplastics are not equal” in terms of their biological effects on aquatic organisms.

## 3. Materials and Methods

### 3.1. Test Organisms

The *D. magna* used in this study were originally sourced from the Korea Institute of Toxicology (KIT), Jinju, South Korea in 2021, and were subsequently reared by Environmental Chemistry & Health Lab (ECH), Pusan National University, Busan, South Korea. The daphnids were cultured under controlled conditions of a 16 h light and 8 h dark cycle at a temperature of 20 ± 1 °C, in accordance with the Organization for Economic Cooperation and Development (OECD) Test Guideline 211 (OECD, 2012) [65]. The organisms were maintained in Elendt M4 medium, with a pH of 7.8 ± 0.2 and water hardness of 250 ± 30 mg L^−1^ CaCO_3_. Exposure experiments were conducted using third-brood female neonates, all of which were less than 24 h old at the time of exposure.

### 3.2. Microplastic Exposure to D. magna

To evaluate the effects of MPs on *D. magna*, each experimental vessel was stocked with 25 neonates less than 24 h old, and experiments were conducted in triplicate. *D. magna* were exposed to two concentrations of PE and PS MPs (100 µg/L and 1000 µg/L) over a 7-day period. A control group, free of MP exposure, was included for comparison. Each vessel contained 500 mL of M4 medium, which was refreshed every 48 h to maintain optimal water quality. The vessels were gently stirred every 8 h to ensure uniform suspension of MP particles.

The selected concentrations span from an environmentally realistic upper-bound (100 µg/L) to an extreme case (1000 µg/L) scenario, enabling a comprehensive evaluation of potential risks and underlying toxicological responses in *D. magna*. Field studies report that MPs in freshwater can reach the mg/L range in polluted areas. For instance, lakes in Texas contained MPs on the order of 1–5 mg/L [66]. Therefore, a number of studies have employed 100–1000 µg/L levels in aquatic toxicity tests. In *D. magna* 21-day assays, ~100 µg/L of MP (1–5 µm PE beads) consistently reduced growth and reproduction [12,67]. Higher exposure of around 1000 µg/L exacerbated these effects and led to organ-level damage in both invertebrates and fish [68,69]. These concentrations are well within or just above ranges for polluted waters, lending ecological relevance to their selection. *Chlorella vulgaris* (a freshwater alga provided by Aquanet, Tongyeong, South Korea) was added daily at a concentration of 5 × 10^5^ cells mL^−1^ to provide a consistent food source.

After the exposure period, seven *D. magna* individuals per replicate were collected for mRNA extraction, and another seven were used to measure global DNA methylation (5-hmC) and hydroxymethylation (5-hmC) levels. All samples were immediately frozen at −80 °C for subsequent molecular analysis. These stored samples were used to analyze gene expression and investigate epigenetic changes in *D. magna*. Ten of the remaining individuals were randomly selected for body length measurements. Subsequently, all individuals, including those measured, were stored at −20 °C for subsequent MP ingestion analysis.

**Figure 5:**
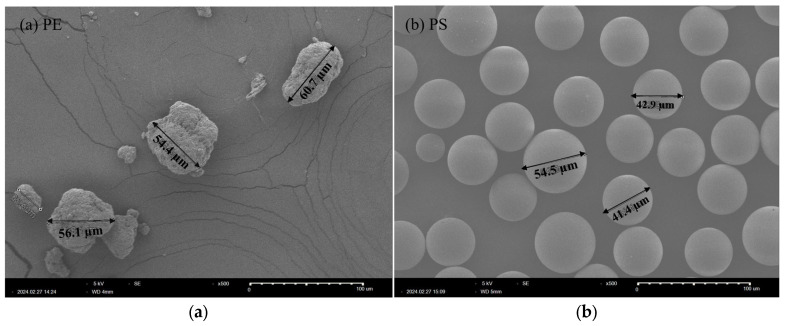
Scanning electron microscope (SEM) images of (**a**) PE MPs and (**b**) PS MPs exposed to *Daphnia magna*.

Standard pristine PE and PS MPs were selected to investigate the polymer-specific effects of MPs. Both types of MPs were chosen due to having similar physical characteristics, which allowed us to minimize variability attributed to shape (refer to Figure 5). PE MPs, with a mean particle size of 40–48 µm, were purchased from Sigma Aldrich, St. Louis, MO, USA (CAS No. 9002-88-74), while PS MPs, with a mean particle size of 38–48 µm, were sourced from the Korea Institute of Analytical Science. Zeta potential (mV) of the MPs was measured using a dynamic light scattering (DLS) analyzer (Zetasizer Nano ZSP, Malvern, UK) to characterize their surface charge. This systematic approach ensured consistency across experiments and allowed for robust analysis of the effects of PE and PS MPs on *D. magna* at molecular, physiological, and behavioral levels.

### 3.3. Gene Expression Analysis (RT-qPCR)

Total RNA was extracted from *D. magna* whole-body tissue samples utilizing a total RNA kit (Cosmogenetech Co., Ltd., Seoul, South Korea). RNA quantity and quality were assessed with a nano-drop spectrophotometer (Thermo Scientific, Waltham, MA, USA). Complementary DNA (cDNA) synthesis was performed using M-MuLV Reverse Transcriptase (Cosmogenetech Co., Ltd., Seoul, South Korea), which was followed by PCR amplification using a Bio-Rad thermal cycler (Bio-Rad, Hercules, CA, USA). Real-time PCR (RT-PCR) was conducted on an AriaMx Real-time PCR System (Agilent, Santa Clara, CA, USA) with the EvaGreen Q Master mix (Cosmogenetech Co., Ltd., Seoul, South Korea). Primers were designed using Primer3plus and based on sequences obtained from National Center for Biotechnology Information (NCBI) database. Primer sets were synthesized by Cosmogenetech (Seoul, South Korea) and optimized for qRT-PCR conditions to ensure high efficiency and sensitivity. All samples were analyzed in triplicate, and mean values were calculated. Control reactions, including those without RT and template, were included to rule out DNA contamination. The housekeeping reference gene *β*-*Actin* was utilized for normalization of gene expression levels.

To evaluate the molecular impact of MP exposure on *Daphnia magna*, we quantified the expression levels of genes associated with stress response and development. A diverse set of biomarker genes was selected to encompass a broad range of biological functions. These include vitellogenin 1 (*v1*), vitellogenin 2 (*v2*), glutathione-S-transferase (*gst*), cytochrome P450 (*cyp*), ATPase Na^+^/K^+^ (*atp*), catalase (*cat*), cuticle protein 12 (*cut12*), ecdysone receptor B (*ecr-b*), and universal stress protein (*usp*).

### 3.4. Global DNA Methylation (5-mc) and DNA Hydroxymethylation (5-hmc) Analysis

Genomic DNA was extracted from the whole-body tissues of *D. magna* using the LaboPass™ Tissue Genomic DNA Isolation Kit Mini (Cosmogenegech Co., Ltd., Seoul, South Korea). DNA integrity and concentration were evaluated using a Nanodrop spectrophotometer. Global DNA methylation and hydroxymethylation levels were assessed using the MethylFlash Global DNA Methylation (5-mC) ELISA Easy Kit and the MethylFlash Global DNA Hydroxymethylation (5-hmC) ELISA Easy Kit (Colorimetric), following the manufacturer’s protocols (Epigentek Group Inc., Farmingdale, NY, USA). In brief, 1–8 μL of DNA (equivalent to 100 ng) was introduced into the assay wells. The DNA was then incubated at 37 °C for a period of 90 min. Capture and detection antibodies were subsequently applied, and absorbance was measured at a wavelength of 450 nm. A standard curve was created using the optical density (OD) values of positive samples, allowing quantification of methylation and hydroxymethylation levels.

### 3.5. Statistical Analysis

All statistical analyses were performed using SAS software (version 9.4; SAS Institute Inc., Cary, NC, USA). Homogeneity of variances and normality of data were evaluated using Levene’s test and the Shapiro–Wilk test, respectively, at a significance level of *p* < 0.05. Differences among multiple treatment groups were analyzed using one-way analysis of variance (ANOVA), followed by Tukey’s post hoc test (*p* < 0.05). For comparisons between two groups, Student’s *t*-test was employed to assess statistical significance (*p* < 0.05).

### 3.6. Microplastic Analysis

#### 3.6.1. Sample Preparation

To identify and quantify MPs accumulated in *D. magna*, 8–11 individuals were collected per vessel in triplicate. The collected organisms were stored in a freezer upon completion of the sample preparation for Fourier Transform Infrared Spectroscopy (FTIR) analysis. To isolate MPs ingested and accumulated by *D. manga*, the samples were digested using a 30% H_2_O_2_ solution at 60 °C to remove organic matter. The digested samples were filtered through Anodisc filters (Al_2_O_3_, 0.02 µm pore size, Whatman, Kent, UK) using a vacuum infiltration device. The filters were stored in a desiccator to remove residual moisture prior to FTIR analysis.

#### 3.6.2. Micro-FTIR Analysis

Identification and enumeration of the MPs collected on the 25 mm Anodisc filter (Al_2_O_3_, 0.02 µm pore size, Whatman, Kent, UK) were conducted using µ-FTIR spectroscopy (Nicolet iN10MX, Thermo Fisher Scientific, Waltham, MA, USA), following previously established protocol. In brief, the filter was divided into 16 predefined sectors which were scanned simultaneously using chemical image mapping to automatically generate high-resolution spectral data from across the entire filter area. The acquired spectra were then matched to the reference database to accurately determine the polymer composition of each particle, allowing for both precise identification and enumeration of MPs. The analysis covered a wavelength range of 1300–4000 cm^−1^, with a collection time of 3 s (16 scans), an aperture size of 60 × 60 µm, and a resolution of 8. Spectra were acquired from the entire area of the 25 mm Anodisc filter where MPs were collected. A background spectrum of the pristine Anodisc filter was collected at the reference position of the sample holder. Analysis was performed on each quarter of the filter area individually, and the data from all four quarters were summed up to present the amount of MPs in the corresponding sample. The acquired spectra from the entire area of the Anodisc filter were correlated with MPs’ reference spectra in the library (e.g., PE and PS), with each being presented with a false-color image based on the matching hierarchy with the corresponding reference spectrum. After examining each individual IR spectrum of the particles with false-color images, particles meeting both the criteria of having a representative IR peak of the reference and having over a 65% matching rate to the reference were identified and counted as MPs.

The limit of detection (LOD) for particle size in micro-FTIR is 20 µm, due to the limit of lateral resolution of the IR source. The limit of the quantity (LOQ) of the micro-FTIR is determined by the optimal conditions for counting MP particles on one filter and the distribution of particles deposited on the filter. Manual counting becomes challenging for particle numbers exceeding a few hundred. Thus, the LOQ is a few hundred particles per filter.

Contamination of MPs in chemicals, filters, and other laboratory consumables was minimized by using glassware for sample storage. Regular blank tests were conducted during the experiment, confirming the absence of MPs.

## 4. Conclusions

This study highlights the impact of MP pollution, specifically PE and PS MPs, on the freshwater zooplankton *D. magna*, demonstrating the potential of MPs to disrupt physiological, epigenetic, and transcriptional processes. The ingestion of the MPs by *D. magna* showed a concentration-dependent pattern, with the PS particles accumulating at higher rates, likely due to their physicochemical properties such as their density and aggregation behavior. Epigenetic analyses revealed significant reductions in the global DNA methylation levels in response to PE exposure, suggesting hypomethylation as a stress response. Although no consistent changes in DNA hydroxymethylation were observed, the variability seen in the PS-exposed groups indicates potential effects that merit further investigation. Transcriptional analysis underscored the complex molecular responses of the studied organisms to MP exposure, including the downregulation of the *v1* gene, associated with a compromised reproductive capacity, and the upregulation of *ecr-b*, which is linked to molting and stress adaptation. These responses reflect adaptive mechanisms employed by *D. magna* to cope with environmental stressors. However, despite these adaptations, the observed reduction in body size across the MP-exposed groups underscores a resource trade-off that may ultimately compromise organismal fitness.

Overall, this study highlights the intricate interplay between microplastic exposure, epigenetic alterations, and gene regulation in aquatic organisms. These findings highlight the pressing need for further research to elucidate the long-term and transgenerational effects of MPs alongside robust efforts to mitigate plastic pollution and its ecological consequences.

## Data Availability

Dataset available on request from the authors.

## Abbreviations

The following abbreviations are used in this manuscript:

MP	Microplastic particle
PE	polyethylene
PS	polystyrene
TRxR	Thioredoxin reductase
SEM	Scanning electron microscope
RT-PCR	Real-time PCR
DLS	Dynamic Light Scattering
FTIR	Fourier Transform Infrared Spectroscopy
